# Physical exercises and weight loss in obese patients help to improve uric acid

**DOI:** 10.18632/oncotarget.22046

**Published:** 2017-10-25

**Authors:** Jun Zhou, Yu Wang, Fan Lian, Dongying Chen, Qian Qiu, Hanshi Xu, Liuqin Liang, Xiuyan Yang

**Affiliations:** ^1^ Department of Rheumatology & Clinical Immunology, The First Affiliated Hospital of Sun Yat-sen University, Guangzhou 510080, China; ^2^ Department of Interventional Oncology, The First Affiliated Hospital of Sun Yat-sen University, Guangzhou 510080, China

**Keywords:** hyperuricemia, BMI, physical activity, waist circumference

## Abstract

**Objective:**

to assess the impact of longitudinal change of the overweight and physical activity on hyperuricemia.

**Methods:**

We performed a retrospective cohort study. Demographic information, clinical features, laboratory findings, body weight and physical exercises pattern were documented.

**Results:**

Altogether 4678 cases of hyperuricemia were enrolled. The median aged males were most affected. Individuals in the middle age had the highest prevalence of being overweight (2501/3382, 74.0%). Middle aged with BMI≥25 kg/m^2^ were more likely to lose weight (963/2807, 34.3%). BMI and waist circumference control helped to reduce serum uric acid. Overweight population was more likely to use urate-lowering or uricosuric medication (3025/3382, 89.4%). Intermediate and heavy activity were associated with bigger SUA improvement. Patients in the age of 35-60 were more likely to do physical exercises than the others.

**Conclusion:**

Being overweight is strongly associated with hyperuricemia. Successful weight control was correlated with significant uric acid reduction. Intermediate to heavy physical activity helps to reduce waist circumference and SUA. In the hyperuricemia population, obese, middle aged men were the most affected, and also the most likely to do more exercises and get their bodyweight back to normal.

## INTRODUCTION

High levels of serum uric acid (UA) has been rapidly increasing in the general population [1, 2]. Accumulating clinical evidence suggests that hyperuricemia is associated with various metabolic disorders [3]. Similar prevalence has been found in China [4]. There are studies indicating that being overweight might be related with different metabolic burden [5].

Obesity [body mass index (BMI) ≥30 kg/m^2^] and central adiposity [waist circumference (WC) ≥80 cm women (WCF), ≥90 cm men (WCM)] are prodromal conditions for metabolic syndrome (MetS) [6, 7]. Previous study showed that intensive physical exercises may prevent athletes from developing metabolic syndrome (MetS). [8]. And some reports suggested that physically active lifestyle would help to improve cardiometabolic disorders. [9, 10].

Recent studies suggested that SUA was a key element underlying excess fat storage [11, 12]. Intracellular UA is an inhibitor for adenosine monophosphate protein kinase (AMPK) activity. When AMPK activity is reduced, fat oxidation is down-regulated, and excess fat is infiltrated [13].

On the other hand, adipose tissue can also produce UA. Adipocytes has xanthine oxidoreductase (XO), and this enzyme is responsible for UA production [14].

Optimal treatment of hyperuricemia is to reach a target level of SUA <6.0 mg/dl. For many years, allopurinol and benzbromarone were the main urate-lowering therapies. Febuxostat, a non-purine XO inhibitor, was launched recently. However, the ability of these therapies to lower SUA was not so promising in some obese patients. Obesity is an important factor of hyperuricemia. Weight control is likely to reduce SUA level and associated costs of medication.

As the prevalence of hyperuricemia correlated with the prevalence of being overweight, the influence of bodyweight control and physically active lifestyle on hyperuricemia treatment remains unclear in China. We carried out the present study to assess the impact of longitudinal change of overweight and physical activity on hyperuricemia.

## RESULTS

Among the 4678 cases of hyperuricemia recorded, the majority were male (3144/4678, 67.2%), and the female accounted for only one third (1534/4678, 32.8%). The median age was 42.3±10.6. Participants at the age of 35-60 years old had the highest prevalence (2807/4678, 60.0%) of hyperuricemia among the three groups. And participants at the age of 18-35 years old had the lowest prevalence (863/4678, 18.4%) of hyperuricemia among the three groups. The patients were all with normal kidney function. They all followed the dietary guidance including alcohol and smoking restriction.

The prevalence of being overweight (3382/4678, 72.3%) was increased in hyperuricemia patients, especially the overweight cases with abnormal waist circumference (2854/4678, 61.0%).

The characteristics of the subjects at baseline were shown in Table 1. Male sex, hypertension, abnormal waist circumference, increased blood measures of fasting plasma glucose (FPG), triglyceride (TG) and decreased high density lipoprotein cholesterol (HDL-C) were strongly correlated with being overweight. Individuals at the age of 35-60 had the highest prevalence of being overweight, and participants >60 years old made up of 53.5% of the normal body weight group (53.5%, 693/1296).

**Table 1:** Demographic characteristics and clinical features of overweight patients with hyperuricemia at baseline

	BMI <25 kg/m^2^ (n=1296)	BMI ≥25 kg/m^2^ (n=3382)	*P*-value
Age (years), Number (%)			
>60	693(45.8)	315(9.3)	<0.05
35-60	306(23.6)	2501(74.0)	<0.05
18-35	297(22.9)	566(16.7)	>0.05
Sex (Male), Number (%)	459(35.4)	2685(79.4)	<0.05
Sex (Female), Number (%)	837(64.6)	697(20.6)	<0.05
Waist circumference, cm, abnormal, Number (%)	45(3.5)	2622(77.5)	<0.05
Elevated blood pressure, Number (%)	576(44.4)	2188(64.7)	<0.05
SBP, mm Hg	137.1±26.4	143.4±29.5	>0.05
DBP, mmHg	68.7±11.5	72.4±13.8	>0.05
Low HDL, *n*	491(37.9)	2653(78.4)	<0.05
Elevated TG, *n*	213(16.4)	2759(81.6)	<0.05
Elevated fasting glucose, *n*	317(24.5)	2036(60.2)	<0.05
Serum UA level, mg/dl	8.05 ± 1.24	8.38 ± 1.37	>0.05

Abnormal waist circumference: ≥90 cm (male) or ≥80 cm (female); Elevated blood pressure : ≥130/85 mm Hg or on hypertension prescription; low HDL : < 1.03 mmol/L (male) or <1.30 mmol/L (female); elevated TG : ≥2.28 mmol/L (within 4h since last meal) and ≥1.7 mmol/L (above 4h since last meal) or on lipid lowering drugs; elevated fasting glucose: ≥ 7.8 mmol/L (within 4 h since last meal) and ≥5.6 mmol/L (above 4 h since last meal) or on glucose lowering prescription; *BMI*: body mass index, *UA:* uric acid, TG: triglycerides, *HDL:* high-density lipoprotein, SBP: systolic blood pressure, DBP: diastolic blood pressure.

Patients with higher BMI had higher SUA levels at baseline.

During the 2 years of follow-up period, we observed the effects of bodyweight change on uric acid levels (Table 2). Participants with BMI≥25 kg/m^2^ were more likely to lose weight when at the age of 35-60 years (963/2807, 34.3%). SUA improvement was associated with BMI and waist circumference reduction. The SUA level was significantly decreased in the ΔBMI from ≥25 kg/ m^2^ to <25 kg/ m^2^ group as compared to the other three groups. Subjects from the normal-to-normal-BMI group showed significant SUA improvement as well. Details of the patients were as follows:

1. Patients in the group of **ΔBMI from BMI <25 kg/m**^**2**^ **to ≥25 kg/m**^**2**^: there were 35 patients (35/178, 19.7%) detected with normal SUA after two years, and all of these patients were on urate-lowering medication. The rest of the patients (143/178, 80.3%) in this group could not achieve normal uric acid level even though they took the urate-lowering medication.
2. Patients in the group of **ΔBMI from ≥25 kg/m**^**2**^ **to ≥25 kg/m**^**2**^: there were 775 patients (775/2163, 35.8%) detected with normal SUA after two years, and none of them lost much weight. They all took the urate-lowering medication. Their improvement of uric acid was not correlated to bodyweight loss.
3. Patients in the group of **ΔBMI from BMI <25 kg/m**^**2**^ **to <25 kg/m**^**2**^: there were 843 patients (843/1118, 75.4%) detected with normal SUA after two years, in which 346 patients didn’t take urate- lowering medication (346/843, 41.0%). [The ratio for patients didn’t take urate-lowering medication with the whole group was 346/1118, 30.9%]. Though their improvement of uric acid was not correlated to bodyweight loss, it seemed that the uric acid level were more easily to be controlled if the patients were not overweight in the whole process.
4. Patients in the group of **ΔBMI from ≥25 kg/m**^**2**^ **to <25 kg/m**^**2**^: there were 1102 patients (1102/1219, 90.4%) detected with normal SUA after two years, in which 311 patients didn’t take urate-lowering medication (311/1102, 28.2%). [The ratio for patients didn’t take urate-lowering medication with the whole group was 311/1219, 25.5%].

**Table 2:** The association between bodyweight change and UA level over 2 years

	ΔBMI from ≥25 kg/m^2^ to <25 kg/m^2^ (n=1219)	ΔBMI from BMI <25 kg/m^2^ to ≥25 kg/m^2^ (n=178)	ΔBMI from BMI <25 kg/m^2^ to <25 kg/m^2^ (n=1118)	ΔBMI from ≥25 kg/m^2^ to ≥25 kg/m^2^ (n=2163)
Age (years), Number (%)				
>60	51(5.06)	16(9.0)	677(60.6)	264(12.2)
35-60	963(79.9)	44(24.7)	262(23.4)	1538(71.1)
18-35	205(16.8)	118(66.3)	179(16.0)	361(16.7)
Waist circumference (cm)				
Baseline	93.57±13.37	78.56±8.35	77.56±8.35	92.16±12.58
Post-intervention	82.45±9.23^*^	86.19±9.69^*^	76.32±7.79	91.44±10.85
SUA				
Base line	9.37±1.18	8.26±1.23	8.53±1.47	9.14±1.41
Post-intervention	6.82 ± 1.25^*^	8.14±1.58	7.05±1.63^*^	8.91±1.59
Patients with normal SUA post intervention,n%	1102(90.4)	35(19.7)	843(75.4)	775(35.8)
Patients not on urate-lowering agents, n%	311(25.5)	87(48.9)	346(30.9)	46(2.1)

*P<0.05, post-intervention versus baseline.

ΔBMI: the change of BMI.

All of the patients who didn’t loss bodyweight, or with increased bodyweight after two years achieved normal SUA by taking urate-lowering agents.

The demographic and clinical parameters were included in the linear regression model. Male sex, bodyweight and waist circumference, elevated triglycerides were considered strongly associated factors with SUA change. As a factor, age did not reach statistical significance.

Patterns of use of urate-lowering agents are shown in Table 3.

**Table 3:** Treatment of hyperuricemia

	BMI <25 kg/m^2^ (n=1296)	BMI ≥25 kg/m^2^ (n=3382)	*P*-value
Use of allopirunol, n	379(29.2)	2031(60.1)	<0.05
Use of febuxostat, n	82(6.3)	189(5.6)	>0.05
Use of benzbromarone, n	367(28.3)	228(6.7)	<0.05
Use of combination urate-lowering therapies, n	35(2.7)	577(17.1)	<0.05
Lifestyle intervention only, n	433(33.4)	357(10.6)	<0.05

The most common doses for allopurinol were 300 mg/day or lower, 40 mg/day for febuxostat and 50-100 mg/day for benzbromarone. A much higher proportion of urate-lowering or combined urate-lowering therapy and a lower proportion of uricosuric medication was found in overweight population. Patients with normal weight was more likely to be recommended lifestyle intervention (433/1296, 33.4%) instead of urate-lowering prescription.

The association between physical activity and SUA are shown in Table 4.

**Table 4:** The association between physical activity and UA

	Light activity (n=2896)	Mediate activity (n=1300)	Heavy activity (n=482)
Age (years), Number (%)			
>60	747(25.8)	234(18.0)	27(5.6)
35-60	1485(51.3)	953(73.3)	369(76.6)
18-35	664(22.9)	113(8.7)	86(17.8)
Sex (Male), Number (%)	1979(68.3)	851(65.5)	314(65.1)
*Waist circumference, N (%)*			
Abnormal baseline	1287(44.4)	1029(79.2)	351(72.8)
Abnormal post intervention	1047(36.2)	464(35.7)^*^	66(13.7)^*^
*BMI≥25 kg/m*^*2*^*, N(%)*			
Baseline	1863(64.3)	1138(87.5)	381(79.0)
Post intervention	1626(56.1)	387(29.8)^*^	43(8.9)^*^
sUA <6.0 mg/dl, N (%)	1566(54.1)^*^	873(67.2)^*^	416(86.3)^*^
post intervention			

*P<0.05, post intervention versus baseline.

Table 4 presents the relationship of activity and SUA level. By comparing the SUA level before and after intervention of medication and physical activity, we found that, all the groups improved, however, the number of patients reach the target of SUA <6.0 mg/dl were significantly different. After we run the regression model, physical activies didn’t reach statistical significance. Then we narrowed down to the patients in the group of ΔBMI from ≥25 kg/m2 to <25 kg/m2, there were 1102 patients (1102/1219, 90.4%) detected with normal SUA after two years, in which 311 patients didn’t take urate-lowering medication (311/1102, 28.2%). In these patents, 259 cases took medium or heavy activities. Our data may not significantly associate intensive physical activities with SUA change directly, but physical activies helped to improve BMI and waist circumference control, and partly contributed to SUA change. In our observation, patients in the age of 35-60 were more likely to do physical exercises than the others.

## DISCUSSION

In this study from south China, we found that being overweight is strongly associated with hyperuricemia. The participants with higher levels of serum uric acid were more likely to be overweight and had abnormal waist circumference. And obese patients were easier to diagnosed hyperuricemia. Consistent with our results, several previous studies have also shown the relationship between overweight and uric acid, and that reduction of BMI could help improve SUA levels [21–24].

In the hyperuricemia population, obese, middle aged men were the most affected in our study. Young female were rarely involved. However, there were substantial shifts in the prevalence of hyperuricemia from middle and aged people to adolescents. Simultaneously, the obese adolescents had higher uric acid than non-obese adolescents. It suggested that we should pay more attention to monitor elevated SUA in young patients and the problem of adolescent obesity.

Interestingly, after 2 years observation, we found that successful weight control, mostly >10kg weight reduction, was correlated with significant uric acid reduction, which was consistent with the report by Dalbeth N, et al., that huge weight loss in severe obesity (BMI > 35 kg/m^2^) cases led to significant SUA reduction, while slight weight loss cause no change of SUA [25]. Zhu Y, et al. also claimed that weight loss of every kilogram was associated with 11% increased odds of achieving the urate-lowering goal, and these associations were independent of other risk factors [24].

In our study, middle age patients were the most likely to be overweight at the baseline, and also the most likely to get their bodyweight back to normal. SUA could be reduced with sustained increasing BMI, but in those patients, much higher proportion of urate-lowering or combined urate-lowering therapy was continued. Therefore, weight loss may be an efficient supplementary therapy to lower SUA concentrations in the obese subjects. But for the hyperuricemia patients with normal bodyweight, weight loss may not have the beneficial effect.

Waist circumference and central body fat distribution was associated with SUA [26].

Overall sedentary behavior is one of the main causes of abdominal obesity. We found that medium to heavy physical activity helps to reduce waist circumference and SUA.

Although the average BMI of Asians is lower, they demonstrate higher fat percentages compared with Caucasians of equivalent BMI [27, 28].

Nutritional education of low purine for hyperuricemia subjects or less food consumption for obesity is popular in China. However, the physical activity education is not so well conducted. Our data suggested that not just food education but also exercises should be put into the lifestyle intervention of hyperuricemia population.

When recommended with change of lifestyle behaviors, the middle age patients had higher compliance than the adolescents and old aged. It’s an interesting phenomenon in the present study.

As the most affected and rapidly increased population of hyperuricemia as well as obesity, they seemed to have more concern than the other two groups of patients. Understanding of these trends helps us to make clinical decisions on different patients.

Nowadays, approximately one-third of the world’s population is obese or overweight [29]. To maintain normal SUA levels, it is important for doctors to not only focus on urate-lowering medicine and nutritional education, but also to pay attention to bodyweight control and exercise. It’s also important to monitor any increase trend of uric acid in obese subjects. Future studies may have to assess whether treatment strategies for the refractory hyperuricemia with obesity need to be targeted weight control as well.

One of the shortcoming of this study is the observational study design. Another shortcoming is the use of uric acid lowering treatment in a vast majority of the population, which reduced the relevance of the findings described. The analysis of the pattern of uric acid control and bodyweight, especially in those didn’t take urate-lowering agents could in part, though not fully, compensated for this limitation.

## MATERIALS AND METHODS

### Study population

The study included 4678 patients over 18 years old with hyperuricemia consecutively admitted to the first affiliated hospital of Sun Yat-sen University, Guangzhou, China, between January 2006 and December 2016.

In all study participants, clinical information was retrospectively investigated, including hyperuricemia and gout history, blood measures of SUA level, glucose, cholesterol, triglycerides, height, weight, systolic blood pressure (SBP), diastolic blood pressure (DBP), smoking status, alcohol consumption, and physical exercises pattern, etc.

Hyperuricemia was defined as: SUA > 7.0 mg/dL for males and SUA > 6.0 mg/dL for females [15, 16]. Three subgroups were categorized by the concentration of SUA: (a) SUA < 7 mg/dL, (b) 7 mg/dL ≦SUA < 9 mg/dL, and (c) SUA ≧ 9 mg/dL [17].

Height and weight (using identical standardized anthropometric scales) were measured twice at each exam, and body mass index (BMI) was calculated as body weight (kg) divided by height squared (m^2^).

BMI was categorized into normal-weight (BMI < 25 kg/m^2^) and overweight (BMI ≥ 25 kg/m^2^). BMI-cutoff of obesity was BMI ≥ 30 kg/m^2^ [18].

Physical activities were recorded and classified by frequency per week, light activity defined as less than 2 times/week with intensity of ≥1 h physical exercises with prominent perspiration or breathlessness, medium activity defined as 3–5 times/week, and heavy activity defined as more than 6 times/week [19, 20].

Exclusion criteria consisted of any of the following factors: impaired renal function or impaired liver function; impaired heart function; patients who were taking drugs that could affect uric acid level, for example, diuretics, glyburide, Aspirin, cyclosporine A, quinolone, etc. were also excluded.

The study was conducted in accordance with the Declaration of Helsinki, and the protocol was approved by the Ethics Committee of the first affiliated hospital of Sun Yat-sen University, Guangzhou, China. (Project identification code: 165661)

### Statistical analysis

Continuous variables were reported as the mean with standard deviation (SD). Chi square tests and independent sample t-tests were used to compare baseline characteristics between participants with normal-weight and overweight. T tests also used to compare waist circumference, SUA levels and BMI between baseline and 2-year post intervention. Linear regression was used for associated factors contributing to SUA change. Covariates were sex, age, total cholesterol, triglycerides, current smoking, physical activity, Hba1c, alcohol consumption, and waist circumference. The variables that were statistically significant by univariate analysis were added into multiple regression analysis. P-values <0.05 were considered statistically significant. The analyses were performed using IBM SPSS 17.0 software.

## Abbreviations

(BMI): body mass index
(WC): waist circumference
(MetS): metabolic syndrome
(AMPK): adenosine monophosphate protein kinase activity
(XO): xanthine oxidoreductase
(SBP): systolic blood pressure
(DBP): diastolic blood pressure
